# Supplementary material for: Asgard archaea defense systems and their roles in the origin of eukaryotic immunity
Source: Nat Commun. 2024 Jul 31;15:6386. doi: 10.1038/s41467-024-50195-2 (PMC11291487; doi:10.1038/s41467-024-50195-2)
Supplement: Supplementary file 3 — Description of Additional Supplementary Files [file 41467_2024_50195_MOESM3_ESM.pdf]

Description of Additional Supplementary Files

File Name: Supplementary Data 1
Description: DefenseFinder output, and descriptive statistics of each dataset analyzed in this work.

File Name: Supplementary Data 2
Description: Information regarding the asVip expression experiment.

File Name: Supplementary Data 3
Description: General information and alignments of the protein structures analysed in this work.

File Name: Supplementary Data 4
Description: Mapping files of the phylogenetic trees.

File Name: Supplementary Data 5
Description: Sequence and structural alignments used to generate the data.
